# Concussion is associated with multiple sclerosis if it occurs before infectious mononucleosis

**DOI:** 10.1093/braincomms/fcag234

**Published:** 2026-08-04

**Authors:** Scott Montgomery, Daniel Smith, Lars Alfredsson, Fredrik Piehl, Tomas Olsson, Ayako Hiyoshi

**Affiliations:** Clinical Epidemiology and Biostatistics, School of Medical Sciences, Faculty of Medicine and Health, Örebro University, Örebro 703 62, Sweden; Clinical Epidemiology Division, Department of Medicine, Solna, Karolinska Institutet, Stockholm 171 77, Sweden; Department of Epidemiology and Public Health, University College London, London WC1E 7HB, UK; Clinical Epidemiology and Biostatistics, School of Medical Sciences, Faculty of Medicine and Health, Örebro University, Örebro 703 62, Sweden; Clinical Trials and Statistics Unit, Cancer Research UK, Sutton SM2 5GP, UK; Institute of Environmental Medicine, Karolinska Institutet, 171 77 Stockholm, Sweden; Centre for Occupational and Environmental Medicine, 13 65 Stockholm, Sweden; Department of Clinical Neuroscience, Karolinska Institutet, 171 77 Stockholm, Sweden; Department of Clinical Neuroscience, Karolinska Institutet, 171 77 Stockholm, Sweden; Clinical Epidemiology and Biostatistics, School of Medical Sciences, Faculty of Medicine and Health, Örebro University, Örebro 703 62, Sweden

**Keywords:** multiple sclerosis, traumatic brain injury, infectious mononucleosis, Epstein–Barr virus

## Abstract

If Epstein–Barr virus is essential for multiple sclerosis (MS) pathogenesis, there may be differences in MS risk for auxiliary exposures that occur before or after Epstein–Barr virus infection. Infectious mononucleosis (IM) during adolescence typically represents the primary Epstein–Barr virus infection, so can be used as a proxy marker, while concussion in adolescence is considered a separate non-essential risk factor for MS. The objective here was to examine if concussion before or after IM during adolescence is differently associated with MS risk. Using national Swedish health registers, among those born from 1980 with follow-up to 2023, we identified a cohort with IM between ages 11 and 20 years among people without a demyelinating disease diagnosis by age 20 years (*n* = 37 432), among whom 174 had a subsequent MS diagnosis. The median age (and interquartile range) at IM was 17.00 (15.52–18.59) years among those without MS and 16.66 (15.15–18.29) years among those with MS. In this cohort, between ages 11 and 20 years, there were 1474 episodes of concussion before IM and 1171 occurring afterwards. Associations between concussion and MS risk were estimated using Cox regression, with age as the underlying timescale and adjustment for age at IM, year of birth (both modelled as continuous measures using restricted cubic splines), sex and county. Notably, only concussion occurring before, but not after, IM was associated with increased MS risk producing adjusted hazard ratios (with 95% confidence intervals) of 2.23 (1.24–4.03; *P* = 0.008) for concussion before IM and 0.85 (0.35–2.09; *P* = 0.726) for concussion after IM. However, there is no statistically significant effect modification for MS with an interaction for concussion before and after IM of 1.60 (0.16–15.60; *P* = 0.685). Our findings suggest that changes in the environment of the CNS caused by concussion facilitate development of MS if present before primary Epstein–Barr virus infection.

## Teaser

Montgomery *et al*. report that concussion before infectious mononucleosis between ages 11 and 20 years is associated with a raised risk of multiple sclerosis. This is consistent with the hypothesis that concussion increases the possibility of an atypical Epstein–Barr virus infection, triggering the process that causes multiple sclerosis.

## Graphical Abstract

**Graphical Abstract:**
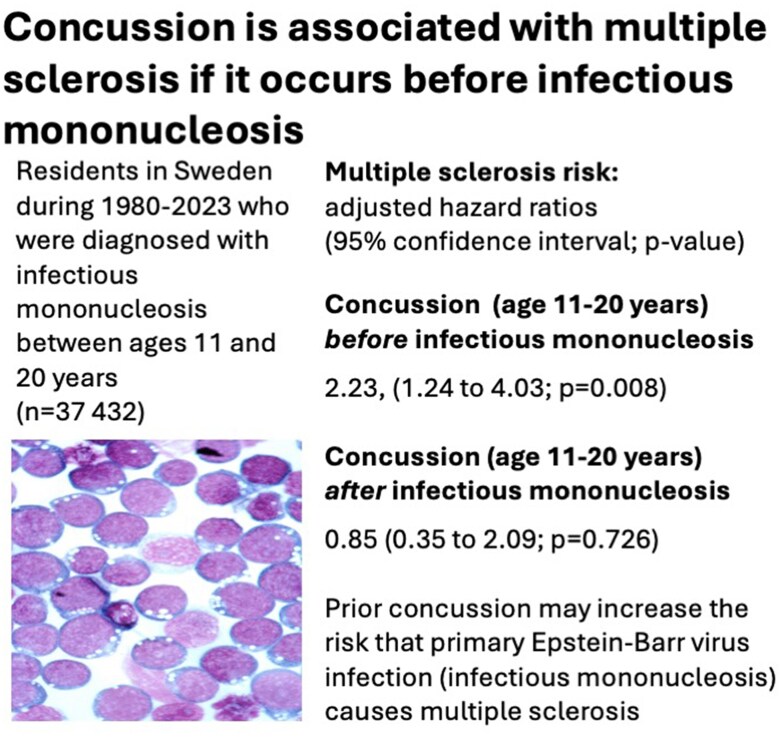

## Introduction

Epstein–Barr virus (EBV) has long been implicated in the aetiology of multiple sclerosis (MS), and more recent evidence suggests it is an essential risk factor for MS to develop.^1,2^ As around over 95% of the adult population are infected by EBV at some point,^3,4^ timing of infection, host genetics and other environmental factors are likely to be involved.^5^ One of the mechanisms put forward for the influence of EBV is molecular mimicry between EBV nuclear antigen 1 (EBNA1)—an essential protein for EBV replication—and host antigens like anoctamin 2, alpha-B crystallin and GlialCAM.^6,7^ Interestingly, Jons *et al.* demonstrated that EBV serological conversion was followed by reactivity to the molecular mimic anoctamin: a rise in the nerve damage marker neurofilament light chain was observed over a period of 10 years or longer.^8^ However, to what degree timing of risk factor exposure in relation to EBV status may also play a role has not been explored in detail. Identifying the precise timing of EBV seroconversion in the general population is challenging, but a first episode of infectious mononucleosis (IM) during adolescence will typically represent the primary EBV infection.^9^

Multiple studies in different populations suggest that traumatic brain injury (TBI) increases the risk of a subsequent diagnosis of MS.^10-12^ Typically, these register-based studies have found that TBI detected during adolescence (ages 11–20 years) is linked with MS that often is diagnosed more than 10 years later.^10^ Most of these events represent milder trauma defined as brain concussion that causes short-term impairment of neurological function not explained by structural injury on routine brain imaging.^13,14^ However, in many cases, long-term alterations in the CNS structure or milieu can be detected with sensitive techniques such as with advanced magnetic resonance protocols or soluble biomarkers.^13,15^ It is also known that brain trauma may lead to release of antigens that cause an adaptive immune response against CNS autoantigens that may influence TBI outcomes.^16,17^ Interestingly, studies across several experimental animal models demonstrate that nerve injury alone, or combined with induction of experimental autoimmune encephalomyelitis (EAE), an animal model of MS, leads to infiltration of immune cells in CNS areas affected by the injury.^18^ That the CNS environment has an impact on MS risk is also supported by genetic association studies identifying risk alleles implicating function of CNS resident cells.^5^ In humans, even non-specific trauma to the CNS, such as following stroke and peripheral nerve damage, leads to expansion of nervous tissue-specific antigens detectable in peripheral blood; and at the same time, affected brain areas become susceptible to infiltration of antigen specific T cells.^19^

This longitudinal cohort study used Swedish register data to assess whether there was a difference in the association with MS with onset after age 20 years, if concussion occurred before or after IM between ages 11 and 20 years among a population of people who had all experienced IM (as a proxy measure of primary EBV infection) in the same age-defined period.

## Materials and methods

### Ethical permission

Ethical permission for this study was obtained from the Swedish Ethical Review Authority (2019-04755, 2023-03585-02 and 2025-03176-01). All data are pseudonymized.

All Swedish residents, born between 2 January 1980 and 30 December 2002, with follow-up to 31 December 2023 and living in Sweden until at least age 21 years, constituted the population from which the analytical sample was drawn, using information from national registers. The Total Population Register provided information on sex, region of residence at birth (24 counties) and dates of birth, death and migration. The Patient Register provided information on inpatient and outpatient hospital diagnoses of MS, other demyelinating diseases of the CNS, IM and concussion using the 9th and 10th revisions of the International Classification of Diseases (ICD). Further information on IM and concussion diagnoses was obtained from primary care data from the county of *Västra Götaland* (∼10% of the Swedish population), with diagnoses identified by ICD-10 codes. IM was identified using 075 for ICD-9 and B27.0 for ICD-10. MS was recorded with codes 340 and G35 for ICD-9 and ICD-10, respectively. All demyelinating diseases of the CNS were identified using codes 340 and 341 for ICD-9 and G04, G35, G36 and G37 for ICD-10. Concussion was recorded as 850 in ICD-9 and S06 in ICD-10.

The main analysis included only individuals with a diagnosis of IM between ages 11 and 20 years (*n* = 37 432), after excluding individuals a demyelinating disease of the CNS diagnosed by age 20 years.

### Statistical analysis

Summary statistics of the variables used in our analyses are presented as counts and percentages for categorical variables, medians and interquartile ranges for continuous variables. Statistical significance of differences was assessed using Pearson’s *χ*² test for categorical variables and the Wilcoxon rank sum test for medians. For the two main exposures of interest, concussion before IM (no, yes) and concussion after IM (no, yes), we quantified the associations with MS using two separate univariable Cox regression models. We then fitted a multivariable Cox regression model, which included both exposures, as well as the pre-specified adjustment covariates. These were sex, county of birth, year of birth and age at first IM. The latter two variables were modelled as continuous using restricted cubic splines with four knots. We also included a shared frailty term for birth county (24 counties) to account for unobserved heterogeneity across regions. Attained age was the underlying timescale, thus adjusting for age effectively. Follow-up was from age 21 years to first diagnosis of MS, emigration or death, whichever occurred first. The proportional hazards assumption was evaluated using the Grambsch–Therneau test and by graphical inspection of scaled Schoenfeld residuals: no violation was identified. Interaction testing was used to assess effect modification for concussion before and after IM.

Instead of using the two exposure variables, a separate model used a single variable with mutually exclusive categories: concussion before IM only, concussion after IM only and concussion both before and after IM. A further analysis dichotomized the concussion variables to characterize concussion in terms of relative timing before or after IM. The four variables produced were concussion up to 1 year before IM, concussion up to 1 year after IM, concussion more than 1 year before IM and concussion more than 1 year after IM. The adjusted analysis included all four variables in the same model, with adjustment for the potential confounding factors.

Bayes factors were calculated^20^ to assist in interpretation of evidence strength. The statistical analysis was performed using R version 4.5.0 particularly using the ‘survival’ and ‘tidyverse’ packages.

## Results

Table 1 shows the characteristics of the study population, all of whom had IM between ages 11 and 20 years and did not have a diagnosis of any CNS demyelinating disease before age 21 years. As expected, there is a greater female predominance among those with MS. On average, those with MS were born a few years earlier than the rest of this population, reflecting the typical ages at diagnosis of MS. The median age at IM was less than half a year younger among those with MS, compared with the rest of the study population.

**Table 1:** Characteristics of people with and without multiple sclerosis who had infectious mononucleosis during adolescence

	No MS (*n* = 37 258)	MS (*n* = 174)	*P*-value
Sex			<0.001
Male	16 290 (44%)	53 (30%)	
Female	20 968 (56%)	121 (70%)	
Birth year			<0.001
Median (interquartile range)	1992 (1988–97)	1989 (1986–92)	
Age at infectious mononucleosis			0.049
Median (interquartile range)	17.00 (15.52–18.59)	16.66 (15.15–18.29)	

Those with a diagnosis of a demyelinating disease before age 21 years are excluded.

MS, multiple sclerosis.

Table 2 shows the associations with MS diagnosed after age 20 years for concussion that occurred before or after IM between ages 11 and 20 years. Concussion before IM is statistically significantly associated with a raised risk of subsequent MS, with a Bayes factor for the adjusted model of 9.524 indicating ‘moderate/substantial evidence’ (range 3–10). Concussion after IM is not associated with MS. These associations are observed before and after adjustment for both measures of concussion, as well as the other covariates: the magnitude of the hazard ratio for concussion before IM increases slightly after adjustment but there is no notable change in the null association for concussion after IM. However, there is no statistically significant effect modification for MS with an interaction for concussion before and after IM of 1.60 (0.16–15.60; *P* = 0.685), indicating the possibility that concussion after IM influences MS risk cannot be excluded. It is notable that adjustment includes age at IM (modelled as a continuous variable), so the association is not explained by differences in age at IM and concussion before or after IM. Table 3 reports a similar analysis, but using a single concussion variable with mutually exclusive categories. Concussion before IM only was statistically significantly associated with MS, and the Bayes factor for the adjusted model indicates ‘moderate/substantial evidence’ (range 3–10). There is also a raised risk for MS associated with concussion before and after IM, but it is not statistically significant. Concussion after IM was not associated with a raised risk of MS.

**Table 2:** Risk of multiple sclerosis associated with concussion before or after infectious mononucleosis during adolescence

	*N* with MS/total *n* (*n* = 37 432)	Unadjusted HR (95% CI)	*P*-value (Bayes factor)	Adjusted HR (95% CI)	*P*-value (Bayes factor)
Concussion before IM					
No	162/35 958	1 (reference)		1 (reference)	
Yes	12/1474	1.99 (1.11, 3.58)	0.021 (4.535)	2.23 (1.24, 4.03)	0.008 (9.524)
Concussion after IM					
No	169/36 261	1 (reference)		1 (reference)	
Yes	5/1171	0.96 (0.39, 2.34)	0.928	0.85 (0.35, 2.09)	0.726

Those with a diagnosis of demyelinating disease before age 21 years are excluded. There are separate variables for concussion before and after IM: some individuals can be exposed in both categories. Adjustment is for the displayed variables as well as sex, year of birth (modelled as a restricted cubic spine with four knots), region (24 counties) and age at IM (modelled as a restricted cubic spine with four knots). Bayes factors are only presented where appropriate.

IM, infectious mononucleosis; HR, hazard ratio; CI, confidence interval.

**Table 3:** Risk of multiple sclerosis associated with concussion before or after infectious mononucleosis during adolescence, modelled in mutually exclusive categories

	*N* with MS/total *n* (*n* = 37 432)	Unadjusted HR (95% CI)	*P*-value (Bayes factor)	Adjusted HR (95% CI)	*P*-value (Bayes factor)
Concussion					
None	158/34 890	1 (reference)		1 (reference)	
Only before IM	11/1371	1.94 (1.05, 3.58)	0.033 (3.270)	2.17 (1.17, 4.00)	0.014 (6.156)
Only after IM	4/1068	0.86 (0.32, 2.31)	0.762	0.79 (0.29, 2.14)	0.642
Before and after IM	1/103	2.63 (0.37, 18.8)	0.335	2.74 (0.38, 19.60)	0.315

Those with a diagnosis of demyelinating disease before age 21 years are excluded. The concussion categories are mutually exclusive and modelled in a single variable. Adjustment is for the displayed variables as well as sex, year of birth (modelled as a restricted cubic spine with four knots), region (24 counties) and age at IM (modelled as a restricted cubic spine with four knots). Bayes factors are only presented where appropriate.

IM, infectious mononucleosis; HR, hazard ratio; CI, confidence interval.

Table 4 shows the association of concussion with MS risk when the duration between concussion and MS is examined. Concussion less than 1 year before IM has a statistically significant and higher magnitude association with MS than seen in the results of the main analysis (Table 2) and the Bayes factor signals ‘moderate/substantial evidence’ (range 3–10) in the adjusted analysis. Concussion more than 1 year before IM has a borderline statistically significant association with subsequent MS and the Bayes factor below 3 indicates ‘anecdotal evidence’ in the adjusted analysis. Neither measure of concussion after IM was associated with MS.

**Table 4:** Risk of multiple sclerosis associated with concussion more or less than 1 year before or after infectious mononucleosis during adolescence

	*N* with MS/total *n* (*n* = 37 432)	Unadjusted HR (95% CI)	*P*-value (Bayes factor)	Adjusted HR (95% CI)	*P*-value (Bayes factor)
Concussion up to 1 year before IM					
No	169/37 112	1 (reference)		1 (reference)	
Yes	5/320	3.42 (1.41, 8.33)	0.007 (10.592)	3.24 (1.32, 8.00)	0.011 (7.416)
Concussion up to 1 year after IM					
No	173/37 140	1 (reference)		1 (reference)	
Yes	1/292	0.74 (0.10, 5.28)	0.763	0.61 (0.09, 4.44)	0.630
Concussion over 1 year before IM					
No	165/36 235	1 (reference)		1 (reference)	
Yes	9/1197	1.86 (0.95, 3.64)	0.070 (1.976)	1.97 (1.00, 3.90)	0.051 (2.424)
Concussion over 1 year after IM					
No	170/36 522	1 (reference)			
Yes	4/910	1.01 (0.37, 2.71)	0.989	0.88 (0.32, 2.38)	0.794

Those with a diagnosis of demyelinating disease before age 21 years are excluded. Adjustment is for the displayed variables as well as sex, year of birth (modelled as a restricted cubic spine with four knots), region (24 counties) and age at IM (modelled as a restricted cubic spine with four knots). Bayes factors are only presented where appropriate.

IM, infectious mononucleosis; HR, hazard ratio; CI, confidence interval.

## Discussion

This national register-based cohort study found that concussion before IM during adolescence is a risk factor for subsequent MS, while concussion that occurred after IM is not, although interaction testing suggests the lack of association for concussion after IM should be interpreted with caution. There is growing evidence that EBV is critical in the aetiology of MS, particularly where primary EBV infection is delayed which increases the risk of IM.^21^ We hypothesize that sequelae of exposures such as concussion can create conditions in the CNS that further increases the risk that an atypical EBV infection results in MS pathogenesis. Concussion up to 1 year before IM had a more notable association with MS than concussion more than a year before IM. After EBV seroconversion, some subsequent environmental exposures may not have the same consequences for MS risk, and this may vary by type of exposure.^22^

Concussion was selected as the exposure that might alter susceptibility to EBV infection as it is practical to identify the precise timing of concussion diagnoses relative to IM. Other known risk factors include smoking, body mass index and vitamin D level,^22,23^ but it is difficult to define precisely the timing of such exposures as this can occur over an extended period. Infectious diagnoses other than IM are another set of potentially important exposures relevant to MS risk, but these may not be independent of each other as one infection can increase the risk of another^24,25^ making it harder to assess potential causation associated with the temporal sequence.

It has been argued that the association of IM with MS is not causal^26^ as human leukocyte antigen (*HLA*) regions such as *HLA-DRB1* and *HLA-DQA1* influence both MS risk and anti-EBV nuclear antigen 1 IgG levels^27-29^; thus, IM may signal MS risk rather than a causal exposure. However, the results from a sibling comparison study are consistent with a causal association between IM and MS.^30^ There is compelling evidence that EBV causes MS: a study reported a 32-fold increased risk of MS following EBV infection, but not other infections, as shown convincingly by a study.^2^ However, some other infections like HHV6A and Rubella have modest magnitude associations with MS risk.^31^ High levels of EBV nuclear antigen-1-specific antibodies and IM are both associated with MS risk independently and synergistically.^32^ A study found further evidence that, before EBV seroconversion, there was no sign of neuroaxonal degeneration (based on levels of neurofilament light chain) indicating that the EBV infection must occur before initiation of MS pathogenesis.^2^ This is consistent with other studies^8^ and our findings, where concussion that occurs before likely EBV seroconversion may influence EBV-related MS risk. The mechanism by which EBV infection causes MS is thought to involve molecular mimicry for EBV transcription factor EBNA 1 and the GlialCAM CNS protein and thus autoimmunity resulting in demyelination,^1^ as well as the involvement of CNS proteins alpha-B crystallin (CRYAB), anoctamin-2 (ANO2), and glial cellular adhesion molecule (GlialCAM).^33^ The delay between EBV infection and diagnosis of MS is consistent with research showing that EBV seroreactivity was present 10 years before the first sign of neuroaxonal injury prior to clinical MS onset.^8^ This damage was associated with anoctamin 2 (a protein that may cross-react) antibodies produced around the time of EBV infection.^8^ Other studies have also demonstrated an average duration of 10–20 years between primary EBV infection and clinical MS onset.^34,35^

It is unlikely that concussion in adolescence is a consequence of prodromal MS, as a previous study demonstrated an association of concussion in adolescence with subsequent MS, but not for non-CNS trauma.^10^ Also, reverse causation seems unlikely as only concussion *before* IM was associated with MS. An interaction between *HLA* type and concussion for MS risk has been demonstrated,^12^ and possible explanations include *HLA*-related influences on recognition or tolerance of CNS antigens resulting from the trauma.^36,37^ Mild TBI may establish an environment in the CNS that facilitates MS pathogenesis when infectious mononucleosis occurs through several putative mechanisms. Concussion can result in immune dysregulation^38^ that could potentially alter the immune response to EBV and the course of the infection. Some sequelae of concussion can persist for months or even years in a subset of patients, with the possibility that a chronic neuroinflammatory state is established.^13,15,39^ Thus, for an extended period after concussion, some characteristics may remain that cause heightened susceptibility to an atypical course for an EBV infection among genetically susceptible members of the population.

There have been secular trends in the incidence of MS in Nordic countries,^40^ so we adjusted for year of birth to improve precision. There is also variation in incidence by region within countries,^40^ so we adjusted for county of birth. This can also account for variation in healthcare and differences in timing of diagnosis between regions. Our use of age as the underlying time scale in Cox regression provided effective adjustment for age and follow-up time. We did not adjust for other MS risk factors as our focus was the relative timing of IM and concussion between ages 11 and 20 years: it is unlikely confounding could notably explain such a *temporal* sequence during this limited age range. We did not adjust for exposures after age 20 years, as we speculate that some environmental exposures after age 20 years, in individuals who already had IM, are less relevant to the initiation of pathogenesis but may only accelerate subclinical disease progression: for example, infections in people with diagnosed MS can cause exacerbations and disease progression,^41^ so there is likely a similar influence on subclinical disease before MS is diagnosed.

A large longitudinal cohort with prospectively recorded diagnoses made it possible to identify the timing of likely first EBV exposure, as well as the relative timing of concussion, but there are some potential limitations. Only ICD-10 identifies IM specifically caused by EBV, but there was insufficient follow-up time and statistical power (few outcome events) to limit our study to IM identified using ICD-10, so we also used earlier ICD versions. However, EBV is the most common cause of IM^42^ and it seems implausible that findings on the relative timing of concussion and IM could be due to other less common causes of IM. It could be argued that the reason for a null finding for concussion after IM is because it is more likely to occur later in adolescence and the age-specific risk for MS associated with concussion may vary. However, as we adjusted for age at IM and this did not alter the results notably, differences in age at concussion relative to IM during adolescence do not explain the findings. This is consistent with a previous study that demonstrated similar risks for MS if concussion during adolescence was before or after age 16 years.^11^ There were somewhat fewer concussion diagnoses after IM and there was no statistically significant effect modification for concussion before or after IM, so it is possible that the lack of association for concussion after IM could be due to limited power. The association of concussion with MS is dose-dependent,^10,12^ but we did not have sufficient power to examine this, so we coded the exposures as concussion yes or no, rather than the number of exposures. The cohort members all had a diagnosis of IM during adolescence, so other patterns of association may exist among those who did not have IM in this age range. This is the first report of these associations, but there were only a relatively small number of outcome events (MS diagnoses) and thus limited statistical power, as our study population was limited to the subset with IM documented in healthcare records. The results are statistically significant and biologically plausible, but a chance finding is always possible, so replication is important.

In conclusion, concussion-related inflammation may alter the CNS environment, increasing the possibility that a subsequent primary EBV infection triggers an inappropriate immune response against the CNS, leading to development of MS among susceptible individuals.

## Supplementary Material

fcag234_Supplementary_Data

## Data Availability

The data used in this study cannot be shared publicly due to regulations under Swedish law. Inquiries about the data used in the present study can be addressed to the corresponding author. Researchers who are interested in Swedish register data can refer to https://dataguiden.se/english. The syntax used for the statistical analysis is available as Supplementary material.
