# Supplementary material for: Concussion is associated with multiple sclerosis if it occurs before infectious mononucleosis
Source: Brain Commun. 2026 Aug 4;8(4):fcag234. doi: 10.1093/braincomms/fcag234 (PMC13435611; doi:10.1093/braincomms/fcag234)
Supplement: Supplementary file 1 [file fcag234_supplementary_data.docx]

global data "(path to data storage)"

use "${data}\filename.dta", clear

* Analytical sample selection

keep if inrange(birthdate_corr, mdy(1,2,1980),mdy(12,30,2002))

*-------------------------------------------------------------------------------*

**Analysis

*-------------------------------------------------------------------------------*

* Survival analysis set-up

stset time, f(event==1)

* Table 1 Descriptive statistics

tab _d //total N

tab kon _d,col chi

tabstat birthyr, by(_d) stat(n mean sd p25 p50 p75)

ranksum birthyr,by(_d)

tabstat age_first_im_11_20, by(_d) stat(n mean sd p25 p50 p75)

ranksum age_first_im_11_20, by(_d)

* Table 2 Survival analysis

*** Panel A

tab concus_before _d

tab concus_after _d

* Unadjusted

stcox concus_before, efron

stcox concus_after, efron

* Adjusted

stcox ///

i.concus_before ///

i.concus_after ///

i.kon ///

birthyr_sp1 birthyr_sp2 birthyr_sp3 ///

age_first_im1 age_first_im2 age_first_im3, shared(birth_lan) efron

*** Panel B

tab conc4 _d

* Unadjusted

stcox i.conc4, efron

* Adjusted

stcox ///

i.conc4 ///

i.kon ///

birthyr_sp1 birthyr_sp2 birthyr_sp3 ///

age_first_im1 age_first_im2 age_first_im3, shared(birth_lan) efron

*** Interaction between concussion before and after IM

stcox i.concus_before i.concus_after ///

i.kon ///

birthyr_sp1 birthyr_sp2 birthyr_sp3 ///

age_first_im1 age_first_im2 age_first_im3, shared(birth_lan) efron

est store a

stcox i.concus_before##i.concus_after ///

i.kon ///

birthyr_sp1 birthyr_sp2 birthyr_sp3 ///

age_first_im1 age_first_im2 age_first_im3, shared(birth_lan) efron

est store b

lrtest a b

* Table 3

tab concus_one_yr_before _d

tab concus_one_yr_after _d

tab concus_more_than_one_yr_before _d

tab concus_more_than_one_yr_after _d

stcox	i.concus_one_yr_before, efron

stcox	i.concus_one_yr_after, efron

stcox	i.concus_more_than_one_yr_before, efron

stcox	i.concus_more_than_one_yr_after, efron

stcox	i.concus_one_yr_before i.concus_one_yr_after ///

i.concus_more_than_one_yr_before i.concus_more_than_one_yr_after ///

i.kon ///

birthyr_sp1 birthyr_sp2 birthyr_sp3 ///

age_first_im1 age_first_im2 age_first_im3, shared(birth_lan) efron
